# Balancing innovation and affordability in anti-obesity medications: the role of an alternative weight-maintenance program

**DOI:** 10.1093/haschl/qxae055

**Published:** 2024-05-02

**Authors:** David D Kim, Jennifer H Hwang, A Mark Fendrick

**Affiliations:** Department of Medicine and Public Health Sciences, University of Chicago, Chicago, IL 60637, United States; Section of General Internal Medicine, Department of Medicine, University of Chicago, Chicago, IL 60637, United States; Department of Internal Medicine and Health Management and Policy, University of Michigan, Ann Arbor, MI 48109, United States

**Keywords:** anti-obesity medication, weight maintenance, health policy, cost-effectiveness, value-based care

## Abstract

Despite remarkable clinical advances in highly effective anti-obesity medications, their high price and potential budget impact pose a major challenge in balancing equitable access and affordability. While most attention has been focused on the amount of weight loss achieved, less consideration has been paid to interventions to sustain weight loss after an individual stops losing weight. Using a policy simulation model, we quantified the impact of a weight-maintenance program following the weight-loss plateau from the initial full-dose glucagon-like peptide 1 (GLP-1) receptor agonists or incretin mimetic use. We measured long-term health care savings and the loss of some health benefits (eg, maintenance of weight loss, improvements in cardiometabolic risk factors, and reductions in diabetes and cardiovascular events). Our model suggested that, compared with continuous long-term full-dose GLP-1 receptor agonists or incretin mimetic drugs, the alternative weight-maintenance program would generate slightly fewer clinical benefits while generating substantial savings in lifetime health care spending. Using less expensive and potentially less effective alternative weight-maintenance programs may provide additional headroom to expand access to anti-obesity medications during the active weight-loss phase without increasing total health care spending.

## Introduction

The launch of highly effective anti-obesity medications promises to mitigate the enormous clinical, equity, and economic toll of the global obesity pandemic. However, their high price and potential impact on long-term health care spending are a formidable barrier to fully reaping the benefits of this scientific breakthrough.

Incretin mimetics (IMs)—a new generation of anti-obesity medications, such as long-acting glucagon-like peptide 1 (GLP-1) receptor agonists—can result in an average of 15%–20% maximum weight loss, improvements in cardiometabolic risk factors, and prevention of secondary cardiovascular events.^1,2^ However, adverse events, including nausea, vomiting, and diarrhea, often lead to medication discontinuation.^3^ Safety issues associated with continued use, such as loss of skeletal muscle mass among older adults and incidence of pancreatitis,^4,5^ are also a concern. The lack of long-term safety data, with the high likelihood of weight regain after medication discontinuation, has questioned the optimal treatment duration, especially if some people will require lifelong treatment to maintain weight loss.^6,7^

The high prevalence of the population clinically indicated for these anti-obesity medications, coupled with their current high acquisition cost, presents major challenges to ensuring equitable access. At present, Medicare prohibits coverage of weight-loss medications, with an estimated obesity prevalence of 41.5% among the 47.4 million enrolled in Medicare Part D plans in 2020.^8^ A prior analysis reported that, if 10% of 19.7 million eligible Medicare beneficiaries used semaglutide at the annual net price of $13 600 after rebates and discounts, it would account for 18.5% of the annual Medicare Part D spending ($26.8 of $145 billion).^9^

Coverage of these agents by commercial insurers is also highly variable and unpredictable. Although almost identical products for diabetes (eg, Ozempic [Novo Nordisk] contain the same active ingredient, semaglutide) are widely covered, the Food and Drug Administration (FDA) has separate indications for weight loss (e.g., Wegovy [Novo Nordisk] maximum dose [2.4 mg] is higher than Ozempic, and Wegovy comes in a single-use injectable pen, instead of multiple doses of the medication). Those payers currently covering these anti-obesity medications often require high levels of consumer cost-sharing and/or have imposed coverage limits based on total spending or duration of use. A growing number of employers and health plans, including the University of Texas system, Ascension, and the North Carolina state health plan, have eliminated coverage of anti-obesity medications, citing skyrocketing costs.^10^ Most recently, one of the largest public universities in the United States issued a new coverage policy on GLP-1 agonists for their employees, limiting their coverage to only up to 24 months. The potential threat of raising premiums was cited as a primary reason.^11^

As obesity and related clinical sequelae disproportionately affect people from racial and ethnic minority populations and those with low socioeconomic status,^12,13^ the limited access and coverage to highly effective obesity-treatment options would further exacerbate health disparities. Thus, identifying patient-centered, clinically driven solutions that more efficiently and equitably allocate resources to managing obesity—while maintaining affordability—is warranted.

### Alternative weight-maintenance program vs continuous IM use

While most of the clinical research and media attention has been focused on the amount of weight loss achieved by various anti-obesity medications, less consideration has been paid to interventions to sustain weight loss after an individual stops losing weight. This timing distinction is essential as the duration (and related expense) of anti-obesity medication use is typically shorter for the weight-loss phase than for the maintenance phase. Thus, once an individual achieves sustained weight loss (ie, achieving maximum weight loss for a certain period, such as a few months) using an GLP-1/IM therapy at recommended doses, an option to switch to an alternative weight-maintenance program—incorporating elements such as lower-cost medications (than full-dose GLP-1s/IMs), a behavioral health program, and nutrition support—has the potential to more efficiently reallocate resources devoted to obesity management efforts.

For example, consider a 44-year-old female patient with class II obesity (baseline body mass index [BMI] of 36 kg/m^2^) and medication-controlled hypertension. Following treatment with full-dose GLP-1s/IMs for 12 months, she achieved a 16% loss in body weight. She has been following a lower carbohydrate eating pattern and exercising at least 5 days per week and notes a significant reduction in hunger since starting GLP-1/IM therapy. She no longer required antihypertensive medications. After her weight has been stable for 2–3 months, she has several options to maintain her weight loss, including (1) continue GLP-1s/IMs at the current full dose or (2) alternative strategies that incorporate behavioral interventions and nutritional support, and a lower cost medication regimen—for example, (2a) less intensive GLP-1/IM therapy (eg, every other week instead of weekly) or (2b) transition to a less expensive anti-obesity medication tailored to the individual patient (eg, phentermine/topiramate if using highly effective contraception). To encourage a switch from full-dose GLP-1s/IMs, patient-centered elements could be incorporated into alternative strategies, such as (1) elimination of cost-sharing for all services included in the alternative program, (2) additional financial rewards for sustained weight loss, and (3) the option for individuals who regain weight to restart GLP-1/IM therapy with no duration limits.

Consideration of a 2-phase framework to obesity management (ie, active weight-loss phase, followed by maintenance of weight-loss phase) allows a potential move away from the current “full-dose GLP-1/IM or nothing” approach to a more personalized weight maintenance approach. Thus, the use of less expensive and potentially less effective alternative strategies focusing exclusively on weight maintenance may substantially reduce medication spending that could be used to expand access to full-dose GLP-1s/IMs for more individuals during the active weight-loss phase without increasing total spending.

Importantly, given a dearth of evidence on the relative clinical effectiveness and aggregate expenditures of an alternative approach compared with continued full-dose GLP-1/IM therapy, a question arises regarding a willingness to potentially sacrifice a proportion of the clinical benefits associated with sustained weight loss associated with long-term GLP-1/IM use. Using a policy simulation model and the concept of decremental cost-effectiveness analysis,^14,15^ this paper aims to quantify what level of savings society would be willing to accept (ie, lower spending on GLP-1/IM drugs) to potentially forgo the health benefits (eg, maintenance of weight loss, improvements in cardiometabolic risk factors, and reductions in diabetes and cardiovascular events) to expand GLP-1/IM access to more people during the weight-loss phase.

## Data and methods

We utilized the Diabetes, Obesity, and Cardiovascular Disease Microsimulation (DOC-M) model—a previously validated and published microsimulation model^14^—to estimate the potential long-term health and economic outcomes of 2 weight-maintenance programs for patients who have reached a weight-loss plateau using recommended GLP-1/IM dosage: (1) continued, long-term full-dose GLP-1/IM treatment and (2) alternative maintenance program (at varying levels of lower cost and clinical effectiveness than continuous full-dose GLP-1/IM treatment).

### DOC-M model

The DOC-M model, a probabilistic and dynamic microsimulation model, projects obesity, diabetes, cardiovascular disease (CVD), and associated complications for guiding population health policy decisions.^14^ Using US population–based transition probabilities, the model tracks a person's annual likelihood of experiencing health events (eg, developing diabetes and CVD) and death based on annual changes in individual-level factors: age, sex, race, BMI, blood pressure, total cholesterol, smoking status, etc. The DOC-M model was extensively validated to demonstrate excellent model performance in predicting changes in population risk in obesity, diabetes, CVD, and all-cause mortality for the overall US adult population and across racial-ethnic groups.^14^Supplement Text A also provides additional details on model development and validation.

The estimated effects of continuous full-dose GLP-1/IM use on BMI and cardiometabolic biomarkers were modeled to change the long-term risk of developing diabetes and experiencing initial/subsequent CVD events based on the built-in US-based risk-prediction models.^16-18^ The DOC-M model applies the US national cause-specific mortality data, stratified by age, sex, and race/ethnicity groups, to project effects on longevity.^19^ The DOC-M model also estimates effects on health-related quality of life (HRQoL) and health care expenditure (HCE) based on changes in individual demographic, socioeconomic, and chronic disease factors. Specifically, obesity-related HRQoL was captured through changes in the presence of obesity-related comorbidities (diabetes, high blood pressure, and CVD events) over time. For obesity-related HCE, both BMI-mediated effects (ie, an additional $89 per 1 unit of BMI increase) and direct effects of obesity-related comorbidities were modeled to estimate the health care cost offsets associated with alternative weight-maintenance programs.

### Continuous full-dose IM use

Data from the Semaglutide Treatment Effect in People with Obesity (STEP) 1 and SURMOUNT-1 trials were used to estimate weight-loss maintenance and long-term improvement in cardiometabolic biomarkers (ie, blood pressure, fasting glucose, cholesterol, triglycerides) and reduction in cardiovascular adverse events.^1,2^ We also accounted for medication-related adverse events (ie, nausea, vomiting, and diarrhea) and post-treatment discontinuation rates (Table 1). We applied a 50% discounted list price of GLP-1/IM drugs (eg, $530 monthly costs for tirzepatide) based on a recent report on implied net price after discounts and rebates.^20,21^ Based on the trial design, our analysis assumes that all patients with continuous full-dose GLP-1s/IMs also continue lifestyle modification, incurring annual costs of $632 with 12 face-to-face behavioral counseling sessions ($53 monthly), which were added to the net price of full-dose GLP-1s/IMs.

**Table 1.** Key model parameters for modeling long-term effects of GLP-1 agonists.

	Lifestyle modifications only (LM)	Semaglutide (SG) + LM	Tirzepatide (TZ) + LM
Effects on weight	−3.1% (95% CI: −4.3% to −1.9%)	−14.9% (95% CI: −13.5% to 15.3%)	−20.9% (95% CI: −21.8% to −19.9%)
Effects on cardiometabolic risk factors (year 1 only)	SBP: −1.2 mmHg DBP: −1 mmHg Fasting glucose: −0.9 mg/dL Cholesterol: −1.1% HDL: 0.2% Triglyceride: −6.3%	SBP: −6.2 mmHg DBP: −2.83 mmHg Fasting glucose: −8.35 mg/dL Cholesterol: −3% HDL: 5% Triglyceride: −22%	SBP: −7.6 mmHg DBP: −4.6 mmHg Fasting glucose: −10.6 mg/dL Cholesterol: −7.4% HDL: 8.2% Triglyceride: −31.4%
Side effects	Not Applicable	Nausea: 44.2% Vomit: 24.8% Diarrhea: 31.5%	Nausea: 24.6% Vomit: 8.3% Diarrhea: 18.7%
AOM discontinuation after year 1	Not Applicable	7%	6.2%
List price of GLP-1	Not Applicable	$16 188 per year ($1349 monthly)	$12 718 per year ($1060 monthly)
Costs of lifestyle modifications	$631.68	$631.68	$631.68
Source	SURMOUNT-1	STEP-1/STEP-2	SURMOUNT-1

Abbreviations: AOM, anti-obesity medication; CI, confidence interval; DBP, diastolic blood pressure; GLP-1, glucagon-like peptide 1; HDL, high-density lipoprotein; SBP, systolic blood pressure.

### Alternative maintenance program

Patients who achieved a sustained weight-loss plateau switched to an alternative program modeled to vary effectiveness in maintaining the maximum weight loss achieved (ie, effectiveness index, ranging from 0 to 1) and monthly cost relative to the best available GLP-1/IM treatment strategy (ie, price index, ranging from 0 to 1). Similar to patients taking continuous full-dose GLP-1s/IMs, we assume that individuals in the alternative program participate in lifestyle modification, incurring costs of $53/month. Based on the current evidence, we assume that the effectiveness of the alternative strategy is relative to the best available weight loss (−20.9% mean weight loss achieved) and maintenance data for full-dose tirzepatide ($530 per month, assuming a 50% discount from the list price).

This “best case” GLP-1/IM approach, which allows for the maximum potential long-term clinical benefit of full-dose GLP-1/IM use, was used as our base case analysis, such that the clinical and cost estimates of the alternative maintenance program would be as conservative as possible. As an additional analysis, we also examined a scenario where the best available GLP-1/IM strategy is based on the weight-loss and -maintenance data for semaglutide (−14.9% mean weight loss achieved) and cost ($675 per month, assuming a 50% discount from the list price) (see Supplement Figure B).

Our model conservatively assumed that the clinical benefits associated with continuous GLP-1/IM use (eg, reduced diabetes and CVD cases, increased longevity, and improved quality-adjusted life-years [QALYs]) would be lost proportionally to the weight regained relative to the maximum weight loss achieved with full-dose GLP-1/IM treatment. For example, if a patient regained 50% of the initial weight lost under the alternative weight-maintenance program (ie, effectiveness index of 0.5), the patient would lose half of the modeled long-term clinical benefits.

### Target population

We modeled the long-term effects of 2 weight-maintenance programs (continuous full-dose GLP-1/IM treatment versus alternative maintenance program) among US adults (20–79 years old) with a BMI of 27–29.9 kg/m^2^ with comorbidities or a BMI of 30 kg/m^2^ or higher. The modeled population was derived from the 2017–2020 (pre-pandemic) National Health and Nutrition Examination Survey (*n* = 4770, representing 134 million adults).

### Decremental cost-effectiveness analysis

The alternative program is projected to lower obesity medication spending (ie, when the price index is <1.0) while sacrificing some benefits (and additional expenditures on obesity-related complications) when it was assumed to be less effective than continuous full-dose GLP-1/IM treatment (ie, when the effectiveness index is <1.0). Thus, we conducted a decremental cost-effectiveness analysis to quantify incremental lifetime health and economic effects of an alternative weight-maintenance program relative to the best available GLP-1/IM treatment strategy.^15^ We measured health effects in differences in QALYs, where negative values indicate QALYs forgone under the alternative program. We also quantified lifetime intervention costs and offsets in health care spending associated with underlying health conditions. As an additional scenario analysis, we assumed the projected long-term health care cost offsets associated with the best available IM strategy were only 50% of the base case analysis. We applied a concept of decremental cost-effectiveness ratios to the clinical and cost results, expressed as dollars saved per QALY forgone, and applied commonly used cost-effectiveness analysis thresholds—$100 000–$150 000 per QALY gained—used in the United States.^22^ We applied a 3% annual discount rate for both costs and QALYs.^23^

## Results

Our policy simulation modeling projected that, compared with continuous long-term full-dose GLP-1/IM use, participation in a less expensive alternative weight-maintenance program following a weight-loss plateau would significantly reduce AOM-related spending and produce minimal reductions in lifetime QALYs over a wide range of relatively lower price and effectiveness indices.

**Figure 1.**
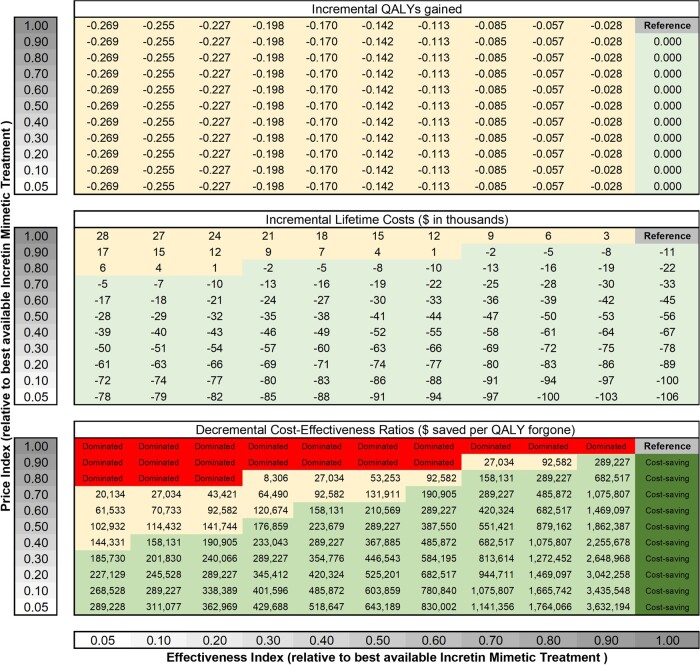
Incremental lifetime health and economic effects of an alternative weight-maintenance program relative to the best available GLP-1/IM treatment. Based on the current evidence, the authors’ analysis assumes that the best available GLP-1/IM treatment follows the weight-loss and -maintenance data for tirzepatide, which is more effective (−20.9% vs −14.9% in mean weight loss achieved) and cheaper ($530 vs $675 monthly costs, assuming 50% discount from the list price) than semaglutide. Patients start with the best available GLP-1/IM treatment for 52 weeks to achieve a sustained weight-loss plateau, then switch to the cheaper, less effective alternative program. “Dominated” indicates that the alternative program is less effective and more costly than the GLP-1/IM strategy. “Cost-saving” indicates that the alternative program is more effective and less expensive than the GLP-1/IM strategy. We applied $150 000 saved per QALY forgone (light green) as a reasonable benchmark for decremental cost-effectiveness ratios. Abbreviations: IM, incretin mimetic; QALY, quality-adjusted life-year.

Figure 1 describes the alternative program's decremental lifetime health and economic effects (relative to the best available GLP-1/IM strategy) over a wide range of effectiveness and price indices, from 0.05 to 1.00. The long-term loss in QALYs ranged from −0.028 QALYs (ie, a loss of 10 days in perfect health), with an effectiveness index of 0.9, to −0.269 QALYs (ie, a loss of 98 days in perfect health), with an effectiveness index of 0.05. Despite fewer long-term clinical benefits, the alternative program is projected to generate substantial savings in net lifetime health care costs (ie, lifetime medication costs net of future health care spending). When the price index is lower than 0.7, the alternative program is estimated to generate net savings in lifetime health care costs, regardless of the effectiveness index. This is because resultant savings attributable to the alternative program are always greater than the potential increases in spending on clinical events due to inferior effectiveness.

For example, a patient in the alternative program with an effectiveness index of 0.3 would regain 70% of the maximum weight loss achieved and lose 70% of the long-term health benefits of reducing diabetes and CVD. In this scenario, the projected long-term loss of QALYs was −0.198 (ie, a loss of 72 days in perfect health), resulting in an additional $20 600 lifetime health care spending on obesity-related adverse events, such as diabetes and CVD events, that would have otherwise been prevented when compared with continuous full-dose GLP-1s/IMs. When we also assume that this alternative program with an effectiveness index of 0.3 would cost 50% of the net cost of the full-dose IM strategy (ie, the price index of 0.5, which we assumed $265 per month [= 0.5 × $530]), the projected savings in lifetime treatment costs due to the lower cost of the maintenance program is $55 700, resulting in a net lifetime savings of $35 100 per person in the program (−$55 700 [saved obesity medication cost] + $20 600 [added spending due to adverse health events]). This combination of reduced effectiveness and cost for this specific program (ie, effectiveness index [0.3] and price index [0.5]) produces a decremental cost-effectiveness ratio of $177 000 (−$35 100/−0.198) saved per QALY lost.

**Figure 2.**
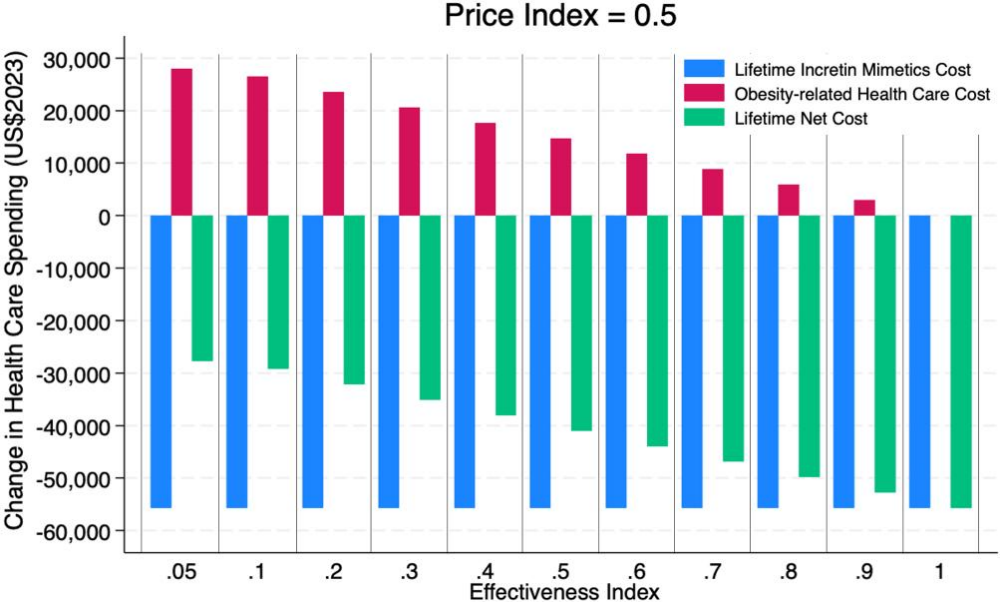
Long-term financial impact of the alternative weight-maintenance program on lifetime health care spending and treatment cost. The figure shows the predicted differences in lifetime health care spending associated with underlying health conditions and treatment costs between enrollment in an alternative weight-maintenance program and continuous long-term full-dose GLP-1/IM use. The price index of 0.5 indicates that the alternative program would cost 50% of the net cost of the full-dose GLP-1/IM strategy, which we assumed to be $265 per month (=0.5 × $530). All costs were discounted at a 3% annual discount rate.

While the relative price and the lifetime cost of the alternative program compared with continuous GLP-1/IM therapy can be accurately estimated, the relative effectiveness is less certain. To address the uncertainty, our model measured lifetime treatment costs saved, additional health care costs incurred from obesity-related complications, and net costs over a wide range of lower effectiveness over a range of price indices. Figure 2 presents these results using a price index of 0.5 over a relative effectiveness range of 0.05–1.0.

Thus, in nearly all scenarios of inferior effectiveness in maintaining weight loss and associated increases in obesity-related clinical benefits, the lower-cost alternative program is projected to generate a small reduction in clinical benefits and a substantial decrease in lifetime costs, yielding improvements in cost-effectiveness. If the best available GLP-1/IM strategy is less effective and more costly (eg, semaglutide) than what we assumed (eg, tirzepatide), the cost-effectiveness of the alternative program would improve, allowing higher price and lower effectiveness indices to meet the same benchmark (Supplement Figure B). In an additional scenario analysis assuming 50% of long-term health care cost offsets used in the base case analysis, an alternative weight-maintenance program becomes more favorable over the continued IM strategy, as the long-term cost offsets would not be as big as those in the base case analysis (Supplement Figure C). The effects were more prominent when the maintenance program was assumed to be much less effective (eg, effectiveness index <0.5) as the potential loss in the long-term savings appeared to be smaller.

## Discussion

The fundamental premise of an alternative weight-maintenance program compared with continuous full-dose GLP-1/IM use is to allow a more efficient and equitable reallocation of resources devoted to managing unhealthy weight. By moving away from an “all or nothing” approach for covering innovative anti-obesity medications, the considerable potential savings incurred from the use of a lower-cost, alternative weight-maintenance program could allow substantially more people—particularly disadvantaged populations that are disproportionally affected by obesity^24^—access to GLP-1/IM treatment to produce optimal amounts of long-term weight loss and reduction in obesity-related sequelae.^25^

Moreover, the available evidence supports that not all individuals who stopped the GLP-1–based anti-obesity medications would regain all the weight they lost during the trial period.^6,7,26^ In the STEP-1 trial extension, almost half of the participants following treatment withdrawal (both semaglutide and lifestyle modification after 68 weeks of treatment) still reported clinically meaningful weight loss of 5% or more from baseline at week 120.^6^ A study analyzing the electronic health records of 20 274 patients who were prescribed semaglutide showed that 56% of patients either maintained or lost additional weight after 12 months since stopping the medication.^26^ Studies have also shown the improved effectiveness of weight maintenance when combining behavioral interventions with anti-obesity medications.^27,28^ In the example scenario where the alternative maintenance program is half the price of continuous full-dose IMs and 30% as effective (ie, patients regain 70% of their weight and lose 70% of the long-term clinical benefit), a net lifetime savings of $35 100 results. The savings from each person switching to this lower-cost maintenance program would fund approximately 6 additional individuals to receive full-dose GLP-1/IM therapy for 1 year. The resulting savings vary considerably over a wide range of the relative price and effectiveness of the alternative program (Figure 1).

Given the greater uncertainty in the effectiveness of the alternative weight-maintenance program (eg, we are even uncertain about how much the use of phentermine/topiramate, followed by the initial GLP-1–based treatment, would maintain the initial weight loss, in contrast to the case when it is being used as a first-line therapy) and the future pricing dynamics, we would not want to set our base case analysis tied to specific medications. However, if the alternative weight-maintenance program includes less effective anti-obesity medications, such as phentermine/topiramate, the effectiveness index would be 0.47 based on the mean percentage of weight lost from baseline: −9.8% (phentermine and topiramate)^1^ vs −20.9% (tirzepatide), while the price index would be 0.57 based on the net monthly cost of $300 (phentermine and topiramate)^29^ vs $530 (tirzepatide). Such an alternative program would result in a long-term loss of 0.142 QALYs (a loss of 52 days in perfect health) while generating a net lifetime savings of $29 800 per person in the program.

Challenges are likely to arise if individuals are offered an alternative maintenance weight-loss program, once they experience an exceptional amount of weight loss produced by full-dose GLP-1/IMs and become aware of reported weight gain upon discontinuation. However, highlighting the potential advantages of a switch that might include (1) reduction in GLP-1/IM side effects and potential long-term adverse effects; (2) lower out-of-pocket cost; (3) financial rewards for sustained weight loss; (4) inclusion of supplemental services, such as nutritional support and exercise programs; and (5) option to restart the GLP-1/IM regimen when needed may overcome the reluctance of some patients to switch.

Finally, we applied the concept of decremental cost-effectiveness analysis to quantify what level of savings society would be willing to accept to forgo an intervention's health benefits.^15^ Although there is no established threshold on what level of savings society would be willing to accept to forgo an intervention's health benefits, published evidence suggests that the “selling price” for a QALY lost would be 1.9 to 6.4 times higher than the “buying price” for a QALY gained.^30^ With a benchmark of $100 000–$150 000/QALY gained as a reasonable threshold of “buying price,”^22^ we could consider much higher thresholds, such as $500 000 saved per QALY lost, for decremental cost-effectiveness ratios. To meet the benchmark at $500 000 saved per QALY lost, the price index of the alternative program is at least 20% lower than the effectiveness index (eg, 20% less effective and 40% cheaper or 40% less effective and 60% cheaper than the full-dose GLP-1/IM strategy).

Our study has some limitations. First, in our modeling, we used the “best case” GLP-1/IM treatment strategy (ie, maximum effectiveness and lowest price) to make differences in health outcomes and cost estimates of the alternative maintenance program as conservative as possible. This rationale notwithstanding, the model did not incorporate the impact of skeletal muscle mass or other unknown long-term adverse effects of the GLP-1/IM strategy, which could make the alternative weight-maintenance program more favorable than the full-dose GLP-1/IM strategy. However, other aspects would make the alternative program less favorable, such as a lack of consideration for the additional benefits of GLP-1/IM use beyond weight-related long-term health benefits (eg, reductions in diabetes-related micro-complications).

Second, the future landscape of GLP-1 agonists—including increased competition, Medicare price negotiation, and generic entry—could potentially lead to further price reductions. However, the future price of the alternative weight-maintenance program may also fall, and the exact magnitude of these changes is difficult to predict. Given this uncertainty, it would be essential to continuously reassess the design and components of alternative weight-maintenance programs. The re-evaluation should consider updated clinical evidence, evolving market dynamics, and the pricing of different strategies. By staying adaptable and responsive to these changes, it would be possible to optimize the effectiveness and cost-effectiveness of alternative programs over time.

Third, our model did not explicitly consider the dynamics of switching therapy (from a weight-loss therapy to a weight-maintenance program) from the perspective of commercial health plans that often face frequent enrollee turnover. Although their share of any long-term cost offsets from downstream health benefits of earlier weight loss could be limited, this effect would be balanced out as these plans eventually exchange enrollees whose high upfront costs of the GLP-1/IM therapy were paid by other health plans.

## Conclusion

The remarkable clinical advances in highly effective anti-obesity medications provide a glimmer of hope to address the global obesity epidemic. However, a major challenge on how to balance equitable access and affordability has emerged. Given the large unmet need and limited resources available, adopting less expensive and potentially less effective weight-maintenance strategies can more efficiently and equitably optimize the clinical benefits of reducing unhealthy weight.

## Supplementary Material

qxae055_Supplementary_Data
