# Supplementary material for: Balancing innovation and affordability in anti-obesity medications: the role of an alternative weight-maintenance program
Source: Health Aff Sch. 2024 May 2;2(6):qxae055. doi: 10.1093/haschl/qxae055 (PMC11138958; doi:10.1093/haschl/qxae055)
Supplement: qxae055_Supplementary_Data [file qxae055_supplementary_data.zip › HASCHOLAR-24-00067.R1 - Online Supplement 2024 04 24.docx]

***Text A. Diabetes, Obesity, and Cardiovascular Disease Microsimulation (DOC-M) Model***

*NOTE: A full description of the DOC-M model development and validation analysis is published elsewhere* (1)*. Here, we provided a brief summary.*

***DOC-M Model overview*:** Programmed in R-4.1.0 (2), the DOC-M model is a probabilistic and dynamic microsimulation model that projects obesity, diabetes, CVD, and their associated complications for guiding population health and nutrition policy decisions. Using US population-based transition probabilities, the model tracks a person's annual likelihood of experiencing health events (e.g., developing diabetes and CVD) and death based on individual-level factors: age, sex, race, blood pressure, total cholesterol, smoking status, and others. Each individual in the DOC-M model can over their lifetime progress through 5 health states: no CVD or diabetes, diabetes without CVD, CVD without diabetes, both CVD and diabetes, and death. Each year they could experience a CVD-related events (first or recurrent CVD, with an option for revascularization). The model also captures the incidence and prevalence of overweight and obesity based on each individual's BMI. (**Figure A**)

**ACC/AHA,** American College of Cardiology/American Heart Association**; ASCVD**, atherosclerotic cardiovascular diseases; **BMI**, body mass index; **BP**, blood pressure; **CHD**, coronary heart diseases; **CVD**, cardiovascular disease; **DM**, diabetes mellitus; **HBP**, high blood pressure; **HDL-C**, high-density lipoprotein cholesterol; **HRQOL**, health-related quality of life; **MEPS**, Medical Expenditure Panel Survey; **HTN**, hypertension; **TC**, total cholesterol

**Figure A1. Conceptual Diagram of the model structure**


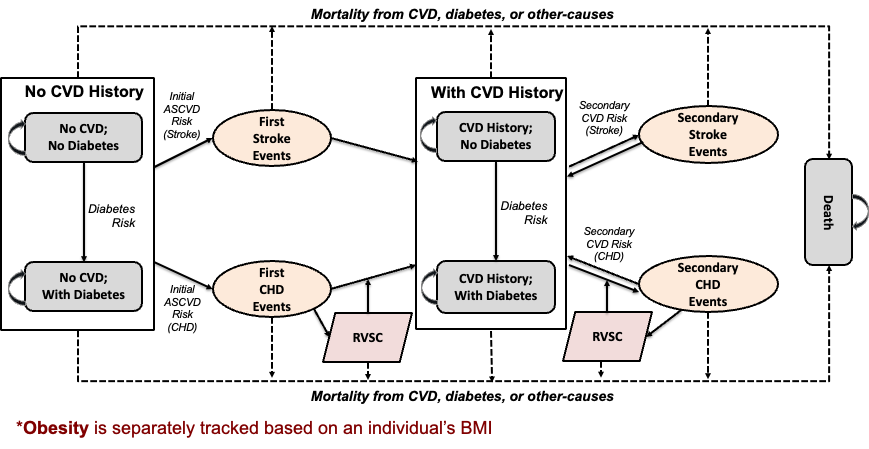


**Major Components of the DOC-M Model:** The DOC-M model applies three US-based validated risk prediction models for developing diabetes, initial CVD events, and subsequent CVD events, respectively (Disease Risk Modules) (3-5). The DOC-M model uses the US national cause-specific mortality data, stratified by age-sex-race/ethnicity groups, available from the CDC Wonder (Mortality Modules)(6). The DOC-M model also incorporates a previously developed health-related quality of life (**HRQOL**) prediction model and an individualized health care cost prediction model for the US nationally representative sample based on demographic, socioeconomic, and chronic disease factors (HRQOL and Cost Modules) (1, 7). Finally, the model incorporates underlying risk factor trends for BMI, total cholesterol, HDL-C, and blood pressure, by estimating the average annual percent change using historical National Health and Nutrition Examination Survey (**NHANES**) data, stratified by age-sex-race/ethnicity groups. The model provides US population estimates by aggregating individual trajectories with their NHANES survey weight.

**Figure A2:** Major model components, key variables, and data sources

**
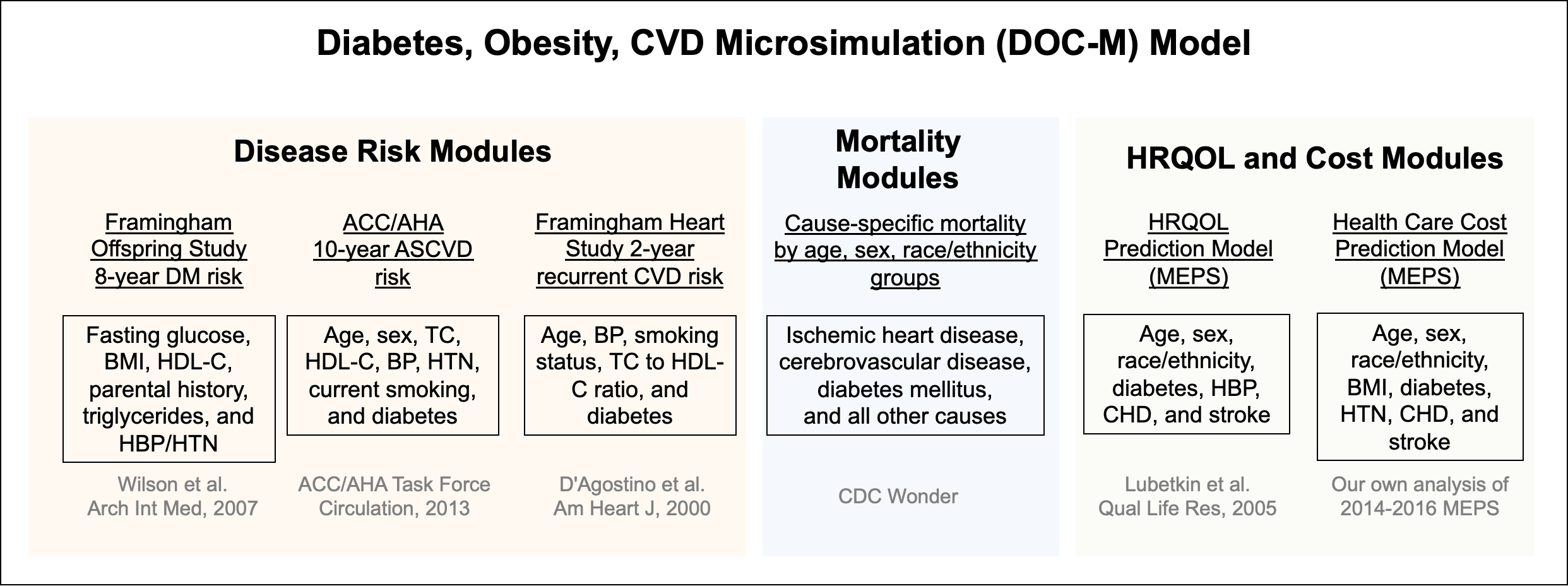
**

***Model validation and calibration*:** We conducted validation analyses for the overall US adult population and three major racial-ethnic adult groups (non-Hispanic white, non-Hispanic black, and Hispanic) in two ways, assessing (1) population-level risk calibration and (2) individual-level risk discrimination for four primary outcomes: obesity, diabetes, ASCVD, and all-cause mortality. Asians and other racial-ethnic minority groups were excluded due to small sample sizes in NHANES data. To assess population-level risk calibration, we first compared the 15-year model-predicted population risk of primary outcomes among the 2001-02 NHANES cohort aged 40-79 years with the observed prevalence of these outcomes in the age-matched population at each NHANES cycle from 2003-2016. Using the observed-to-expected ratio and calibration plot analysis, we assessed whether predictions were systematically too low or too high (8-10). We also assessed how well the model differentiated individuals who experienced (or did not) primary events in the Multi-Ethnic Study of Atherosclerosis prospective cohort, a racially-ethnically diverse sample of 6,814 asymptomatic men and women aged 45-84 years at baseline (11). Using the MESA baseline 2000-2002 data, we estimated each individual’s predicted risk by repeating their simulations 1,000 time to determine predicted likelihood of developing obesity and/or diabetes at Year 9 and CVD and all-cause mortality at Year 14. Then, we assessed risk discrimination with receiver operating characteristics curves that compared individual predictions to observed events documented through MESA follow-up (12, 13).

***Model performance:*** Our validation analyses demonstrated excellent model performance based on population-risk calibration and individual-risk discrimination for all primary and secondary outcomes for the overall US adult population and three racial-ethnic groups, albeit with greater uncertainty among non-Hispanic Black and Hispanic adults due to a smaller sample size. Our 15-year mean prediction from the NHANES 2001-2002 cohort fell within the 95% confidence interval of the actual observed national prevalence for all primary (obesity, diabetes, CVD, and all-cause mortality) and secondary outcomes (cause-specific mortality), both overall and within three racial-ethnic groups. Calibration plot analysis demonstrated excellent performance for all primary and secondary outcomes overall and within each of the three racial-ethnic groups. In most cases, Brier scores (i.e., mean squared error) fell below 0.0004, with the largest Brier score being 0.0012 for obesity among Hispanic adults. In the MESA cohort, the model showed strong individual risk-discrimination overall and within each of the three racial-ethnic groups, for all of the primary outcomes. The estimated c-statistics were 0.85-0.88 for diabetes, 0.93-0.95 for obesity, 0.74-0.76 for CVD history, and 0.78-0.81 for all-cause mortality.

**Figure B. Incremental Health and Economic Effects of an Alternative Weight Maintenance Program relative to Semaglutide over a Patient’s Lifetime**
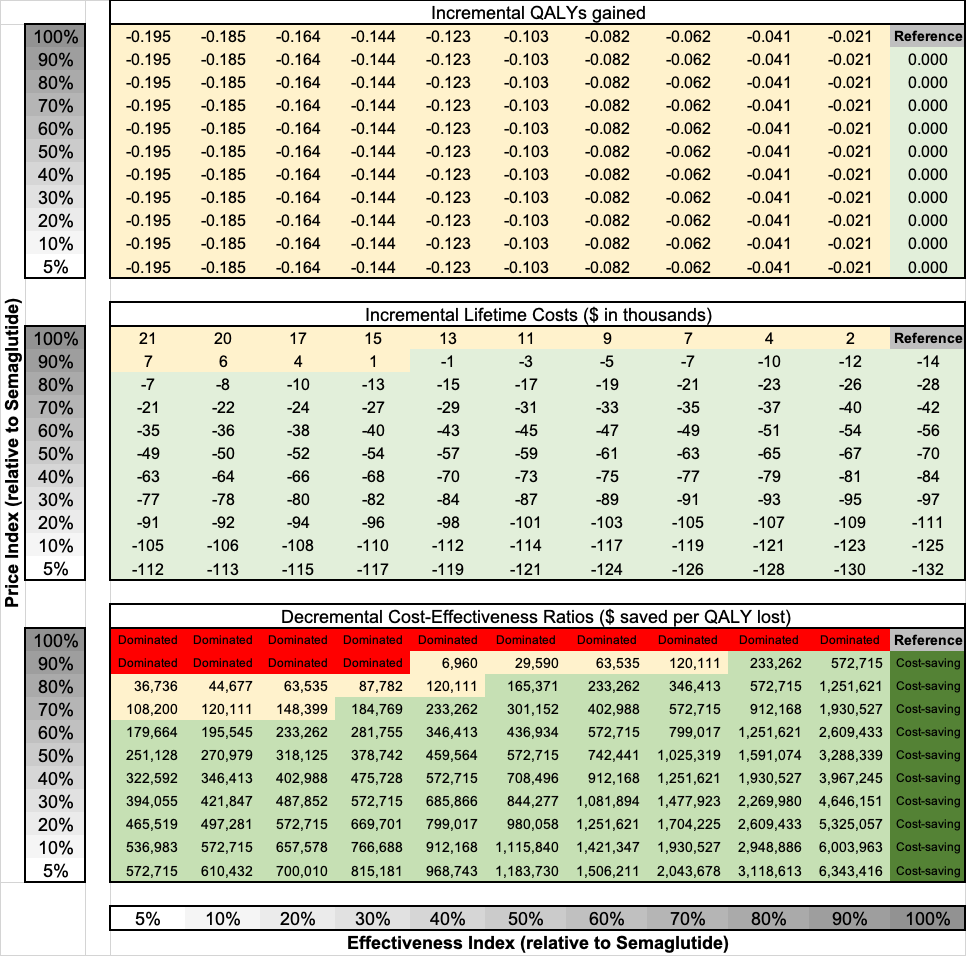


**Note**: Based on the current evidence, the analysis assumes that patients start with semaglutide for 52 weeks (14.9% in mean weight loss achieved, with $675 monthly costs, assuming a 50% discount from the list price), then would switch to the cheaper and less effective alternative program. “Dominated” indicates that the alternative program is less effective and more costly than semaglutide. “Cost-saving” indicates that the alternative program is more effective and less expensive than semaglutide. We applied $150,000 saved per QALY forgone [light green] as a reasonable benchmark for decremental cost-effectiveness ratios.

**Figure C. Sensitivity Analysis: The Impact of Applying 50% of the Long-term Health Care Cost Offsets Associated with the Best Available IM Strategy (Base Case Analysis)**


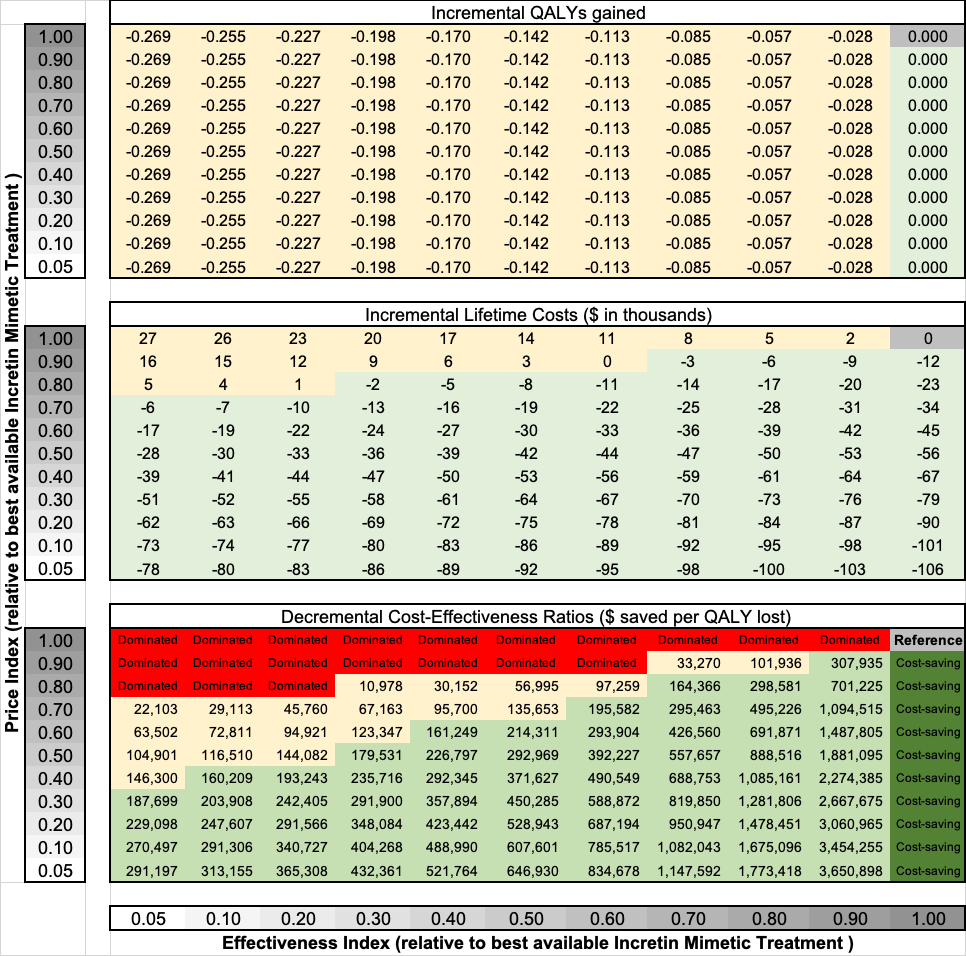


**References**

1. Kim DD, Wang L, Lauren BN, Liu J, Marklund M, Lee Y, et al. Development and Validation of the US Diabetes, Obesity, Cardiovascular Disease Microsimulation (DOC-M) Model: Health Disparity and Economic Impact Model. Medical Decision Making. 2023;43(7-8):930-48.

2. R Core Team. R: A language and environment for statistical computing2016 February 28, 2019. Available from: <https://www.R-project.org/>.

3. Wilson PW, Meigs JB, Sullivan L, Fox CS, Nathan DM, D'Agostino RB, Sr. Prediction of incident diabetes mellitus in middle-aged adults: the Framingham Offspring Study. Arch Intern Med. 2007;167(10):1068-74.

4. Goff DC, Jr., Lloyd-Jones DM, Bennett G, Coady S, D'Agostino RB, Gibbons R, et al. 2013 ACC/AHA guideline on the assessment of cardiovascular risk: a report of the American College of Cardiology/American Heart Association Task Force on Practice Guidelines. Circulation. 2014;129(25 Suppl 2):S49-73.

5. D'Agostino RB, Russell MW, Huse DM, Ellison RC, Silbershatz H, Wilson PW, et al. Primary and subsequent coronary risk appraisal: new results from the Framingham study. Am Heart J. 2000;139(2 Pt 1):272-81.

6. Centers for Disease Control and Prevention. Underlying Cause of Death 1999-2016 Atlanta, GA2018 [Available from: [https://wonder.cdc.gov/wonder/help/ucd.html#](https://wonder.cdc.gov/wonder/help/ucd.html).

7. Lubetkin EI, Jia H, Franks P, Gold MR. Relationship among sociodemographic factors, clinical conditions, and health-related quality of life: examining the EQ-5D in the U.S. general population. Qual Life Res. 2005;14(10):2187-96.

8. Steyerberg EW, Vickers AJ, Cook NR, Gerds T, Gonen M, Obuchowski N, et al. Assessing the performance of prediction models: a framework for traditional and novel measures. Epidemiology. 2010;21(1):128-38.

9. Van Calster B, Nieboer D, Vergouwe Y, De Cock B, Pencina MJ, Steyerberg EW. A calibration hierarchy for risk models was defined: from utopia to empirical data. J Clin Epidemiol. 2016;74:167-76.

10. van Geloven N, Giardiello D, Bonneville EF, Teece L, Ramspek CL, van Smeden M, et al. Validation of prediction models in the presence of competing risks: a guide through modern methods. BMJ. 2022;377:e069249.

11. Bild DE, Bluemke DA, Burke GL, Detrano R, Diez Roux AV, Folsom AR, et al. Multi-Ethnic Study of Atherosclerosis: objectives and design. Am J Epidemiol. 2002;156(9):871-81.

12. Pandya A, Sy S, Cho S, Alam S, Weinstein MC, Gaziano TA. Validation of a Cardiovascular Disease Policy Microsimulation Model Using Both Survival and Receiver Operating Characteristic Curves. Med Decis Making. 2017;37(7):802-14.

13. Harrell Jr FE. Regression modeling strategies: with applications to linear models, logistic and ordinal regression, and survival analysis: Springer; 2015.
